# Cryptococcal Choroid Plexitis and Non-Communicating Hydrocephalus

**DOI:** 10.7759/cureus.8512

**Published:** 2020-06-08

**Authors:** Kyle P O'Connor, Panayiotis E Pelargos, Camille K Milton, Jo Elle G Peterson, Bradley Bohnstedt

**Affiliations:** 1 Neurosurgery, University of Oklahoma Health Sciences Center, Oklahoma City, USA; 2 Pathology, University of Oklahoma Health Sciences Center, Oklahoma City, USA; 3 Neurosurgery, Indiana University, Indianapolis, USA

**Keywords:** cryptococcus neoformans, endoscopic, biopsy, infection, hydrocephalus

## Abstract

*Cryptococcus neoformans *is a fungus that commonly invades the central nervous system. While the choroid plexus, the site of the blood-cerebrospinal fluid barrier, serves as one potential entry point for the pathogen, disease involvement of the choroid plexus itself remains a very rare manifestation of *Cryptococcus *infection. In cases in which choroid plexus involvement blocks cerebrospinal fluid flow, obstructive hydrocephalus may occur. Here we report the case of a 63-year-old woman who presented with choroid plexitis causing obstructive hydrocephalus at the foramen of Monro. Endoscopic biopsy confirmed *Cryptococcus neoformans,* and the patient was successfully treated with amphotericin, flucytosine, and fluconazole. With proper recognition and treatment of this pathology, patients can fully recover from this condition.

## Introduction

*Cryptococcus neoformans* is a fungus that can invade the central nervous system (CNS), typically causing meningitis, encephalitis, ventriculitis, or meningoencephalitis [1]. Most commonly, *Cryptococcus* invades the body through the respiratory system and resides in the lungs. This infection may then spread via a hematogenous route to infect the brain [2]. While *Cryptococcus* represents the most common fungal infection of the CNS, choroid plexitis is a rarely reported manifestation of the disease [1,3]. Proper treatment of cryptococcal choroid plexitis involves a multidisciplinary approach, involving specialists in the fields of neurosurgery, neurology, pathology, and infectious disease/mycology. Here we report the history, diagnostic evaluation, and management of a patient presenting with choroid plexitis and obstructive hydrocephalus secondary to cryptococcal infection.

## Case presentation

**Figure 1:**
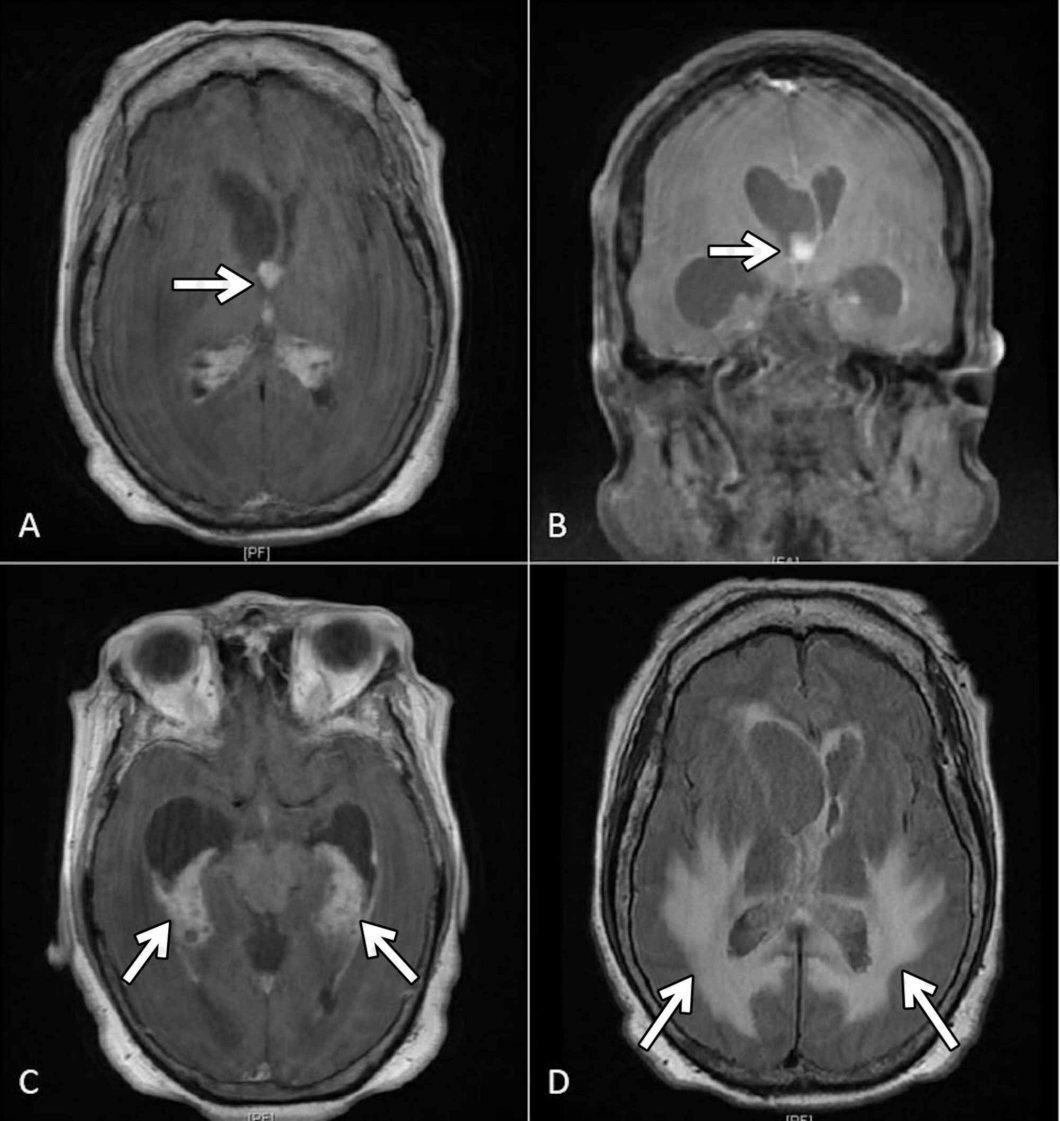
MRI Findings of Choroid Plexitis (A) Axial T1-weighted post contrast MRI and (B) coronal T1-weighted post contrast MRI demonstrating a hyperintense nodularity at the foramen of Monro (arrows). (C) Axial T1-weighted post contrast MRI demonstrating involvement of the choroid plexus within the temporal horn of the lateral ventricle (arrows). (D) Axial section of T2-weighted FLAIR MRI demonstrating transependymal flow (arrows). FLAIR, Fluid-attenuated inversion recovery.

A 63-year-old woman with a history of alcohol use disorder, end-stage liver disease, and mycobacterial tuberculosis presented with one-month history of progressive diffuse weakness and altered mental status. The patient’s condition had progressed to the point of being unable to care for herself or make decisions. She was accompanied by her sister, the patient’s designated medical power of attorney, who provided informed consent for all tests, interventions, and treatments. T1-weighted post-contrast MRI and a T2-weighted MRI were obtained, which demonstrated an enhancing mass within the right foramen of Monro and findings suggestive of choroid plexus inflammation manifesting with right-sided trapped temporal horn syndrome. No abnormal signal was observed within the brain parenchyma, and the basal cisterns were preserved in appearance. MRI findings are shown in Figure 1.

**Figure 2:**
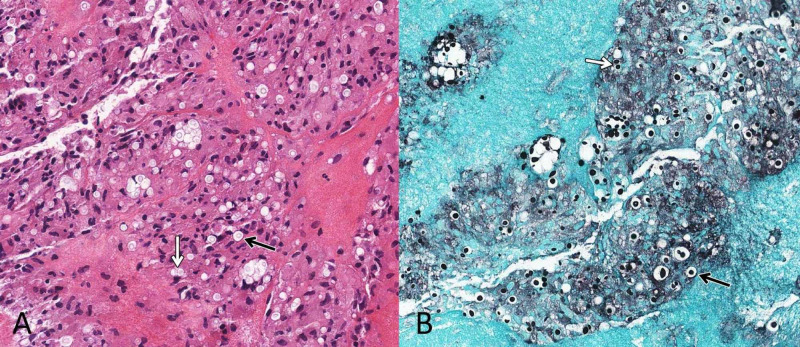
Hematoxylin and Eosin and Methenamine-Silver Staining of Biopsy Sample Hematoxylin and eosin (A) and Gomori methenamine-silver (B) stains (both X20) demonstrate the presence of *Cryptococcus*. Encapsulated C*ryptococcus* yeasts are indicated by the black arrows and budding yeasts are indicated by the white arrows.

**Figure 3:**
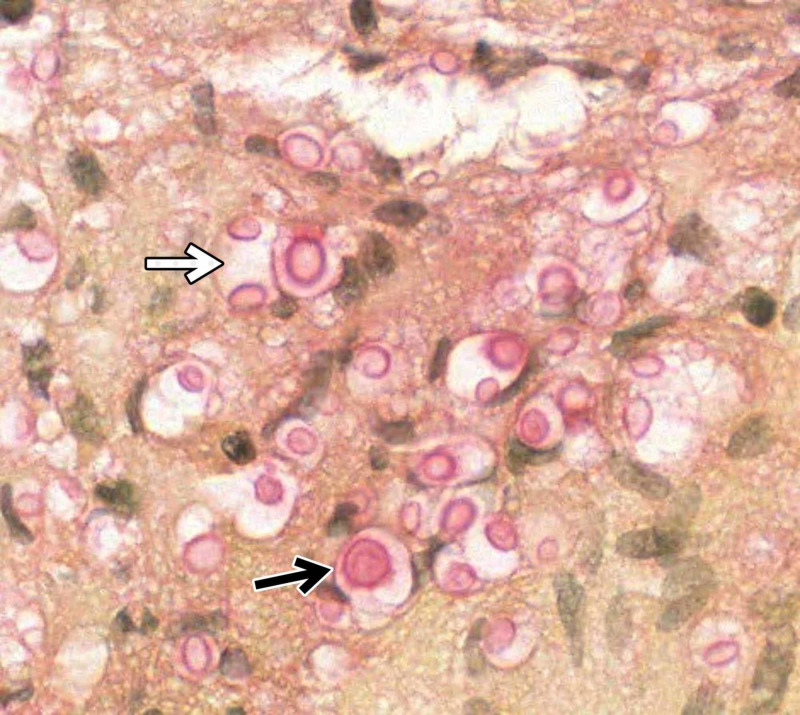
Mucicarmine Staining of Biopsy Sample Mucicarmine staining (X40) demonstrates the presence of a mucicarmine-positive (pink) capsule. An encapsulated *Cryptococcus *yeast is indicated by the black arrow and budding yeasts are indicated by the white arrow.

Blood cultures acquired at patient presentation demonstrated positivity for *Cryptococcus neoformans*. Subsequently, an acute decline in the patient’s mental status was observed and the patient was taken emergently to the operating room for endoscopic biopsy and septum pellucidotomy. Under image guidance, an endoscope was extended through a burr hole and dural incision at the right Kocher’s point to the right frontal horn. Both the foramen of Monro mass and what appeared to be inflamed choroid plexus were biopsied. Using image guidance and anatomic landmarks, a small hole was made through the septum pellucidum using endoscopic graspers. The endoscope was used to expand the opening and was then passed into the left frontal horn. Cerebrospinal fluid (CSF) encountered on this side of the opening was observed to be yellow-green in color. Following biopsy and septum pellucidotomy, an external ventricular drain (EVD) was placed in the right frontal horn. Pathologic evaluation of the biopsied tissue demonstrated an abundance of budding yeasts with mucicarmine-positive capsules, most suggestive of *Cryptococcus* species, but not excluding differentials of *Candida* or *Histoplasma* species. CSF drawn intraoperatively as well as from the EVD was culture positive for *Cryptococcus neoformans*. In addition, a latex agglutination test of the CSF was positive and a serum cryptococcal antigen titer of 1:128 was suggestive of cryptococcal choroid plexitis in this setting. Pathology findings are shown in Figures 2, 3.

The patient was started on induction therapy IV liposomal amphotericin 5 mg/kg daily and flucytosine 1,500 mg q6h once the diagnosis was made. The altered mental status improved over the next few days. Ten days after placement of the EVDs, the patient underwent a clamp trial. The CT scans demonstrated unchanged ventricle size, so both EVDs were removed. The patient had no complications with regards to the choroid plexitis and was discharged 18 days following admission with a peripherally inserted central catheter (PICC) line in place for continuance of the amphotericin. The patient completed four weeks of amphotericin and flucytosine. The patient was prescribed consolidation therapy with fluconazole 800 mg for four weeks followed by a maintenance dose of 200 mg to be continued lifelong. Lifetime maintenance antifungal therapy was deemed necessary due to the patient’s multiple medical comorbidities and her poor likelihood of surviving a repeat cryptococcal infection of the CNS.

## Discussion

Background

*Cryptococcus neoformans* causes fungal infections most commonly in the lungs, and the infection can spread to the CNS by a hematogenous route. When it affects the CNS, it can cause meningitis, encephalitis, ventriculitis, or meningoencephalitis. Our report demonstrates a rare cause of *Cryptococcus* producing choroid plexitis that is rarely reported [4-7]. When the choroid plexus is inflamed, the result is rarely symptomatic; however, it can potentially obstruct the flow of CSF leading to hydrocephalus and resultant symptomatology.

Clinical manifestations

When *Cryptococcus* affects the CNS, it can cause headache, fever, cranial neuropathies, altered mentation, lethargy, memory loss, and signs of meningeal irritation. Symptoms usually develop over weeks; however, patients can present more acutely. Compared to immunocompetent patients, immunodeficiency leads to higher CSF burden and quicker onset of symptoms. While cryptococcosis mainly affects the lungs and CNS, it can also infect the skin, prostate, eyes, bones, and joints [3].

Magnetic resonance imaging

MRI findings are dependent on the way that the infection is housed within the CNS. Ventricular involvement causes abnormal ependymal enhancement along the wall of the ventricles and commonly suggests choroid plexitis. If choroid plexitis blocks the flow of CSF, hydrocephalus will result. Parenchymal involvement shows abnormally enlarged perivascular spaces in the basal ganglia on T2-weighted images or it can show lesions on T1-weighted images. Meningeal involvement demonstrates an enhancing leptomeningeal thickening [8]. 

Histology and serology

The polysaccharide capsule is an important virulence factor that allows this organism to evade the immune system. First choice for diagnostic testing is *Cryptococcus *antigen detection because of its high sensitivity. Other diagnostic tests include mucicarmine, India Ink, enzyme-linked immunosorbent assay (ELISA), and latex agglutination. The benefit of latex agglutination is that it can determine which of the four serologic types the patient is infected with (serotypes A-D). *Cryptococcus neoformans* serotype was not assessed in the presented case.

*Cryptococcus neoformans *is divided into three subtypes: var. grubii (serotype A), var. neoformans (serotype D), and serotype AD (a hybrid of serotypes A and D). A recent study suggested that serotypes A and D, compared to serotype AD, are more likely to present as disseminated disease and are less likely to resolve after treatment with amphotericin B plus flucytosine or fluconazole alone [9].

Treatment

Proper treatment involves CSF drainage via an EVD, an external third ventriculostomy (ETV), or a lumbar drain if hydrocephalus is present. Primary treatment involves amphotericin B (0.7-1 mg/kg/day) plus flucytosine (5-FC) (100 mg/kg/day) for two weeks. If 5-FC is intolerant or unavailable, patients can be treated with amphotericin B deoxycholate (AmBd) 0.7-1 mg/kg/day, liposomal amphotericin B (L-AMB) 3-4 mg/kg/day, or amphotericin B lipid complex (ABLC) 5 mg/kg/day for four to six weeks. Other treatment options for cryptococcal infections of the CNS include AmBd (0.7-1 mg/kg/day) plus fluconazole (800 mg/day) for two weeks, fluconazole (fluconazole ≥800 mg/day, preferably 1,200 mg/day) plus 5-FC (100 mg/kg/day) for six weeks, fluconazole (800-2,000 mg/day, preferably 1,200 mg/day) for 10-12 weeks, or itraconazole (200 mg BID) for 10-12 weeks [3].

Other considerations

When choroid plexitis is present, it is important to rule out involvement of the adjacent brain parenchyma, leptomeninges, and cisternal spaces. These lesions can be seen using MRI and are low intensity on T1-weighted images and high intensity on T2-weighted images. T2-weighted images can also be used to look for enlargement of the perivascular spaces, which also suggest infection with *Cryptococcus*.

Other important causes of choroid plexitis includes tuberculosis, cytomegalovirus, as well as bacteria and parasites. Non-infectious causes include sarcoidosis, xanthogranulomas, and rheumatoid nodules [1].

## Conclusions

We have presented a rare case of *Cryptococcus* causing choroid plexitis with hydrocephalus diagnosed by endoscopic biopsy and serology. Diagnosis is best made using *Cryptococcus* antigen testing on CSF and treatment involves high potency and long-term antifungal medications.
